# Tribological behaviors of laser textured surface under different lubrication conditions for rotary compressor

**DOI:** 10.1038/s41598-023-32490-y

**Published:** 2023-04-03

**Authors:** Shaopeng Ding, Huijun Wei, Ouxiang Yang, Liying Deng, Di Mu

**Affiliations:** 1 State Key Laboratory of Air-Conditioning Equipment and System Energy Conservation, Zhuhai, 519070 Guangdong China; 2 Gree Electric Appliances, Inc. of Zhuhai, Zhuhai, 519070 Guangdong China; 3 Guangdong Key Laboratory of Refrigeration Equipment and Energy Conservation Technology, Zhuhai, 519070 Guangdong China

**Keywords:** Engineering, Mechanical engineering

## Abstract

Tribological behaviors of laser textured surface with elliptical dimples were experimentally compared with that of the smooth one under different lubrication conditions, including the poor-oil, rich-oil and dry lubrication. The lubrication regime was analyzed with the increasing operating load by ring-on-ring tribological tests. Finally, the performance impact of rolling piston rotary compressor with textures fabricated on the thrust surfaces was investigated. Results show that the tribological improvement strongly depends on lubrication condition. With the increase of applied loads under rich-oil and poor-oil lubrication, the effect of micro dimple promotes the critical load transforming lubrication regime, and expands the range of hydrodynamic lubrication, meanwhile maintains a similar minimum of friction coefficient as the smooth surface but enhances wear resistance. However, it is reverse to increase the friction coefficient and surface wear for the textured surfaces under dry lubrication. The compressor performance can be improved significantly by laser surface texturing with a 2% reduction of friction power consumption and a 2.5% enhancement of energy efficiency ratio.

## Introduction

Improving efficiency is an eternal topic for compressors used in air conditioners, especially with the increased awareness of global warming, and a highly efficient compressor is strongly demanded to reduce the power consumption. With the use of low global warming potential (GWP) refrigerants, the lubrication regime and operation condition in compressors will be also further worsened. The friction loss and wear failure of sliding surfaces become a main obstacle to improve performance and extend life, especially for the rolling piston rotary compressor with many sliding parts such as the sliding bearings, thrust bearing, rotating crankshaft and roller, reciprocating slide and so on.

Laser surface texturing (LST) by fabricating the regular micro-pattern on surfaces has been confirmed theoretically and practically to improve the higher load-carrying capacity and the lower friction coefficient and surface heat in hydrodynamic bearings, mechanical seals, cylindrical face rings or piston rings^1–4^. This provides a substantial way to improve the tribological behaviors of friction couple.

Compared with the protective surface coating^5,6^ and structural optimization^7^ as the current main methods, the LST just artificially structures surface topography to control the lubrication regime instead of complex processing and difficult design. The micro dimples as the common textured pattern was first carried out the mechanism study of tribological benefits by theoretical modeling and experimental observation^8,9^. It is concluded that surface micro-dimples can boost the additional hydrodynamic pressure of the convergent viscous fluid between the relative sliding components, thus expand the range of hydrodynamic lubrication. As a benefit, the micro dimples, served as micro-hydrodynamic bearings, can maintain surface separation and non-contacting operation under rich-oil condition. Besides, these micro dimples also can be acted as lubricant micro-containers to supply oil source under mixed or poor-oil lubrication, or micro-traps for wear debris to prevent further abrasive wear under dry sliding contact^10^.

Currently, the significant popularity on the studies of micro-dimpled textures was forced on the geometrical optimization (dimple depth^10^, dimple diameter^11^, area density^12,13^), pattern comparison (circle^14^, elliptical^15^, triangular^16^, diamond shape^17^ and flat^18^, spherical^19,20^, sloped concave or convex^21^) and arrangement influence (inclination angle^17^, slender ratio^22^, distribution location^21^) with the purpose of best friction reduction and wear resistance, especially under full-oil or rich-oil lubrication. Overall, the optimal elliptical dimples show a stronger hydrodynamic effect with a 26.3% increase of load-carrying capacity than the circle one^18^ due to fluid cumulative effect in the dimple length direction, and its friction coefficient can be reduced by 10–20% compared with other dimple patterns^23^. So the elliptical dimples were selected and analyzed in this paper.

However, the optimal geometries with tribological benefits highly depend on operating environments^24^ and lubrication conditions^25,26^, which may lead to conflicting conclusions and make a difficult industrial application. For example, by pin-on-disc tests under mixed lubrication condition, Podgornik^25^ carried out the tribological investigation on the effectiveness of surface textures to point that the textures resist sliding and increase friction in his cases with sliding speed from 0.015 to 0.45 m s^−1^ and contact pressure of 1 MPa. But, Liew^27^ showed the friction coefficient of dimpled surface is 11–24% lower than that of the non-textured one in his cases with sliding speed from 0.5 to 7.8 m s^−1^ and contact pressure from 0.08 to 0.3 MPa, and Braun^28^ also showed a friction reduction of up to 80% can be obtained for the optimal diameter at the sliding speed of 0.5 m s^−1^ and contact pressure of 3 MPa. Though the promising results have been proved by a large number of theoretical and experimental studies, the vast majority is based on own operating condition under single lubrication mode. But for industrial applications, especially for the rolling piston rotary compressor in air conditioners, the oil supplies are ever-changing including poor-oil and even dry lubrication, and not merely rich-oil condition. This also significantly affects the lubrication regimes of friction faces from hydrodynamic to mixed or boundary lubrication. So it is lack of the comparability of dimpled optimization and influence on friction and wear properties under the same operation parameters with different lubrication conditions. So in this paper, the tribological behaviors of laser textured surface were compared experimentally under different lubrication conditions with the same operating parameters.

In compressor applications, the textured surfaces have also been paid more attention gradually due to its advantages of friction reduction and anti-wear enhancement, but the published researches still remain inadequate. Nagata^29^ compared three textured patterns on thrust surfaces of reciprocating compressors to show that the coefficient of performance has 1.4% higher and the friction loss has 20–60% lower. Mishra^30^ investigated the tribological behaviors of LST under poor-oil lubrication for scroll compressor within refrigeration ambient, which shows that surface texturing shows significant tribological improvements and largely independent of the type of lubricant or refrigerant. Through the influence of dimpled structures has been investigated in reciprocating or scroll compressor, its applicability and advantage on tribological properties of rotary compressor is not clear.

So, the tribological behaviors of laser textured surface with elliptical dimples were analyzed experimentally under different lubrication conditions including the poor-oil, rich-oil and dry lubrication. The friction coefficients and wear topographies were compared with the smooth one with the increasing operating load. The lubrication regime and wear mechanism were also analyzed. Finally, the performance impact of rolling piston rotary compressor with textures fabricated on the thrust surfaces was investigated.

## Tribological experiments

### Samples

**Figure 1:**
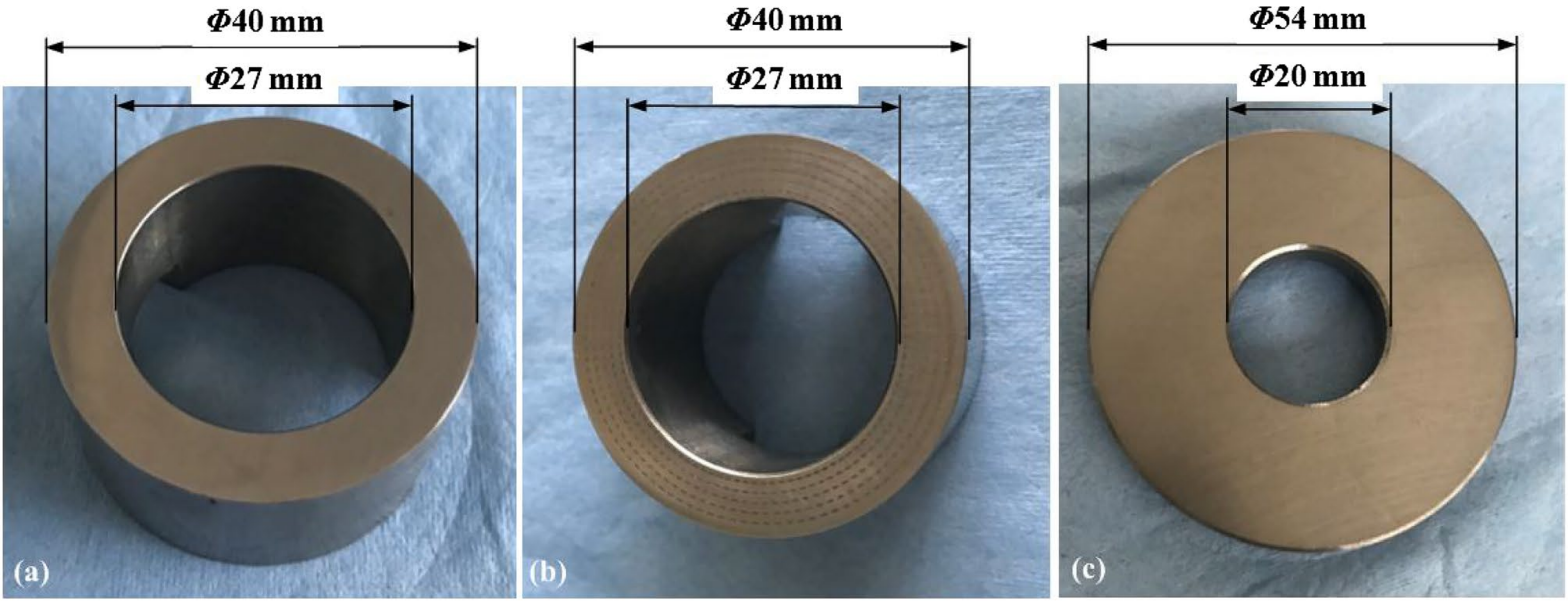
Photographs and dimensions of test samples: (**a**) rotational sample without textures; (**b**) rotational sample with textures; (**c**) stationary sample.

**Figure 2:**
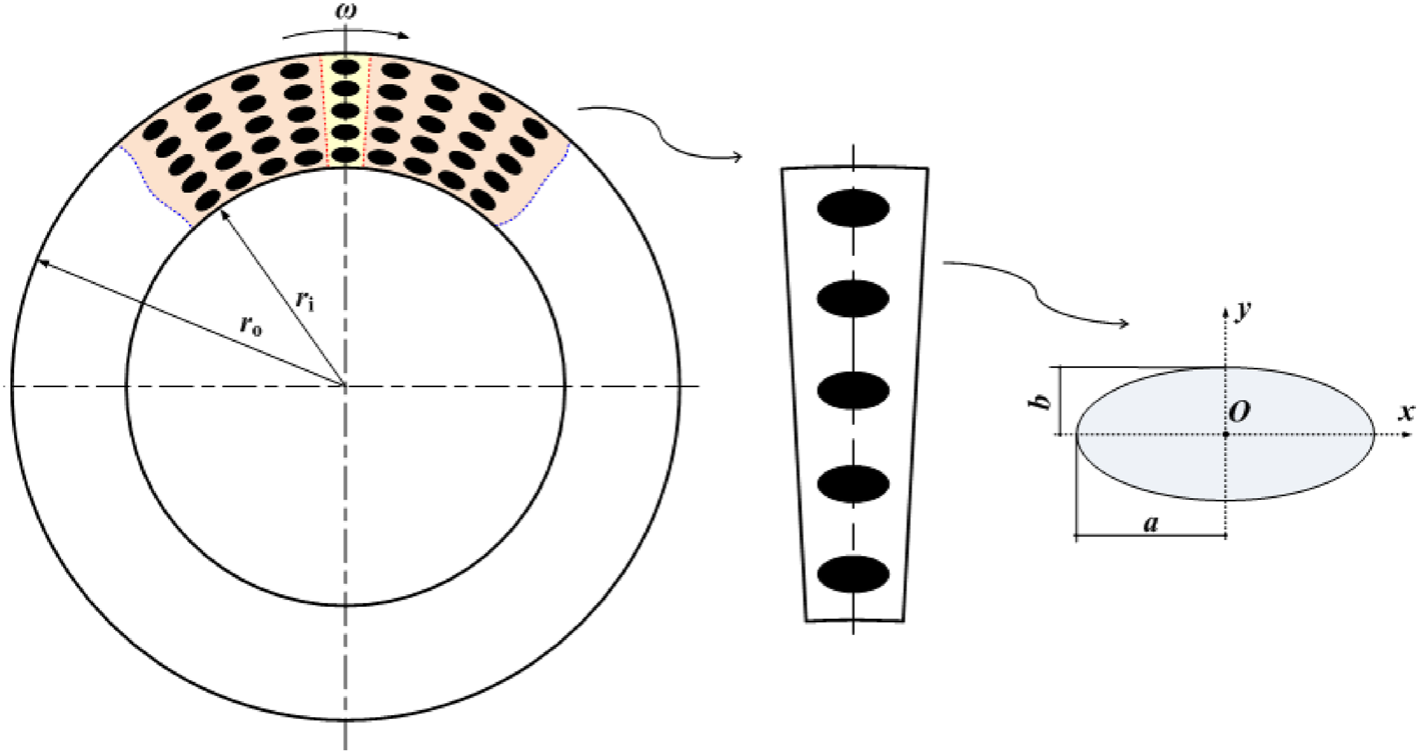
Geometry of textured surface with elliptical dimples.

The experimental tests were carried out on a ring-on-ring friction couple as shown in Fig. 1. The rotor, namely the upper one with the internal radius *r*_i_ = 13.5 mm, external radius *r*_o_ = 20 mm and thickness of 24 mm, was made of JIS FC300 cast iron coming from the roller of rolling piston rotary compressor. The elliptical dimples shown in Fig. 2 were processed by laser surface texturing (LST) with a certain designed depth *h*_d_ = 5 μm, and its major axis was parallel to shear velocity direction. Two extra geometrical parameters were defined to describe the distribution characteristic of elliptical dimple, including the dimple area density, *S*_p_ = *n*_θ_*n*_r_*ab*/(*r*_o_^2^ − *r*_i_^2^), representing the percentage of the summation of dimpled area to lubrication area, and the slender ratio, *λ* = *a*/*b*, representing the ratio between the major radius and minor radius. The detailed meaning and dimension of each variable was listed in Table 1.

**Table 1:** Meanings and dimensions of different geometrical parameters of elliptical dimples on rotor surfaces.

No	Designed depth *h*_d_/μm	Slender ratio *λ*	Dimple area density *S*_p_	Dimple numbers		Dimple patterns
				Circumferential direction *n*_θ_	Radial direction *n*_r_	
1#	–	–	–	–	–	Smooth
2#	5.0	2	9.5%	100	5	Ellipse

**Figure 3:**
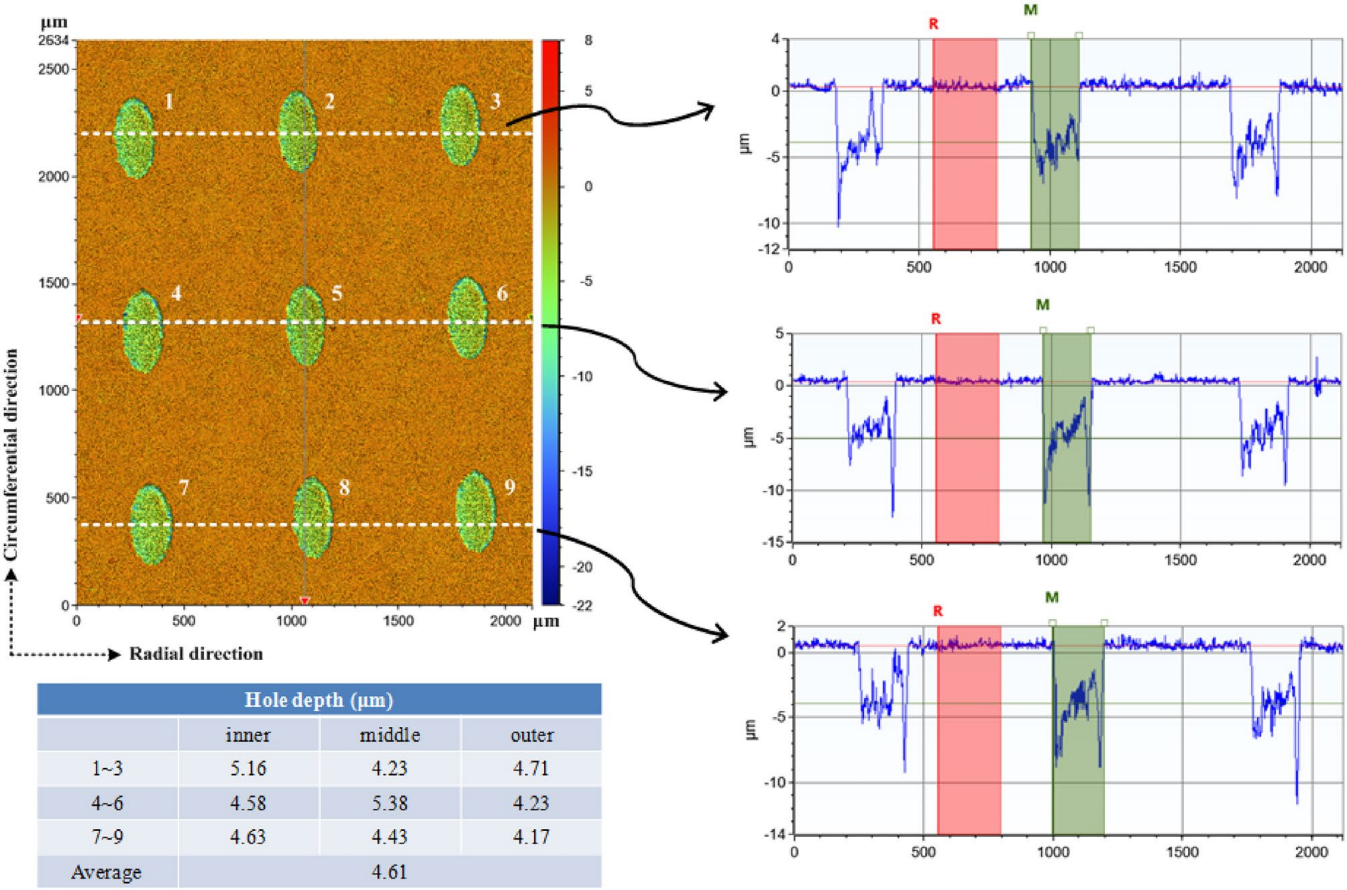
Topographies of the elliptical dimpled surface.

Before laser processing, the original rotor surface was polished with the roughness about Ra < 0.2 μm, then was cleaned in an ultrasonic cleaner with acetone and alcohol, and dried in oven. The micro-dimpled textures were fabricated by fiber optical laser from HGTECH LSF20 with a wavelength 1064 nm. The processing parameters contained the laser power of 7 W, scanning speed of 800 mm s^−1^, frequency of 80 kHz and 3 overscan. After fabricating by fiber optical laser, the polishing process was repeated to remove ridges or bulges around the dimples owning to metal melting by thermal diffusion. The roughness on non-textured surface was controlled in Ra < 0.2. The topographies were measured by a white-light interfering 3D profilemeter supported by BRUKER Contour GT-K shown in Fig. 3. The measured depth was 4.61 μm instead of the designed value of 5 μm.

The stator, namely the bottom one with the internal radius of 10 mm, external radius of 27 mm and thickness of 7 mm, was made of HT250 cast iron, a common bearing material in rolling piston rotary compressor. The stator surface was also polished with the roughness about Ra < 0.2 μm.

### Testing parameters

**Figure 4:**
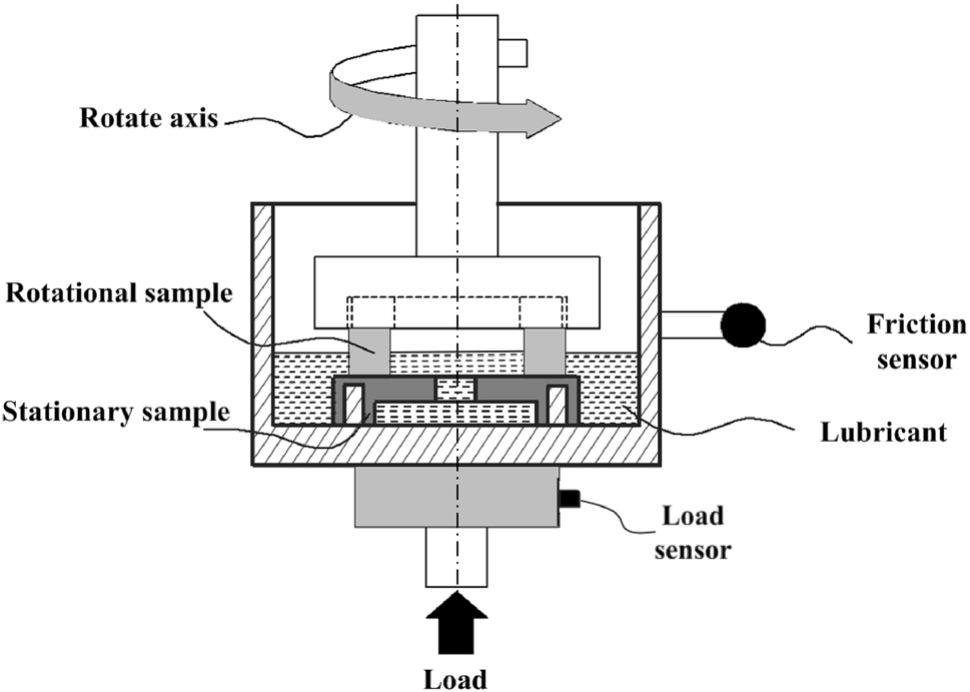
Schematic of test rig.

Tribological behaviors of the laser textured specimens were compared with the smooth one under different lubrication conditions and operating loads by a MMW-1A tribometer. The test rig was shown in Fig. 4 with the ring-on-ring mates under ambient pressure. The upper textured rotor was driven by a rotating motor with a certain rotational speed of 1500 r min^−1^. The bottom smooth stator was clamped in the fixed support, and sustained the vertical applied load. Table 2 lists the operating conditions. Each group test was conducted for 60 min, and was repeated at least three times. The friction torque and friction coefficient were measured. While the testing was completed, the wear topographies on frictional surfaces were presented by SEM analysis (FEI Quanta 250).

**Table 2:** Operating conditions during tests.

Item	Symbol	Dimensions and data
Pressure in inner diameter	*P*_in_	101,325 Pa
Pressure in outer diameter	*P*_out_	101,325 Pa
Rotational speed	*ω*	1500 r min^−1^
Applied load	*F*	100–700 N
Lubrication condition	–	Poor-oil lubrication
		Rich-oil lubrication
		Dry lubrication

The influences of different lubrication conditions on tribological performances were compared including the poor-oil, rich-oil and dry lubrication. To the rich-oil lubrication, during the whole running, the rotor and stator were submerged into the lubricant (FV50S) with a certain volume of 100 mL. To the poor-oil one, the lubricant was evenly applied to the friction interface before startup, but there was no subsequently lubricant supplying. To the dry lubrication, any lubricant was not been provided.

### Friction and wear characteristics

**Figure 5:**
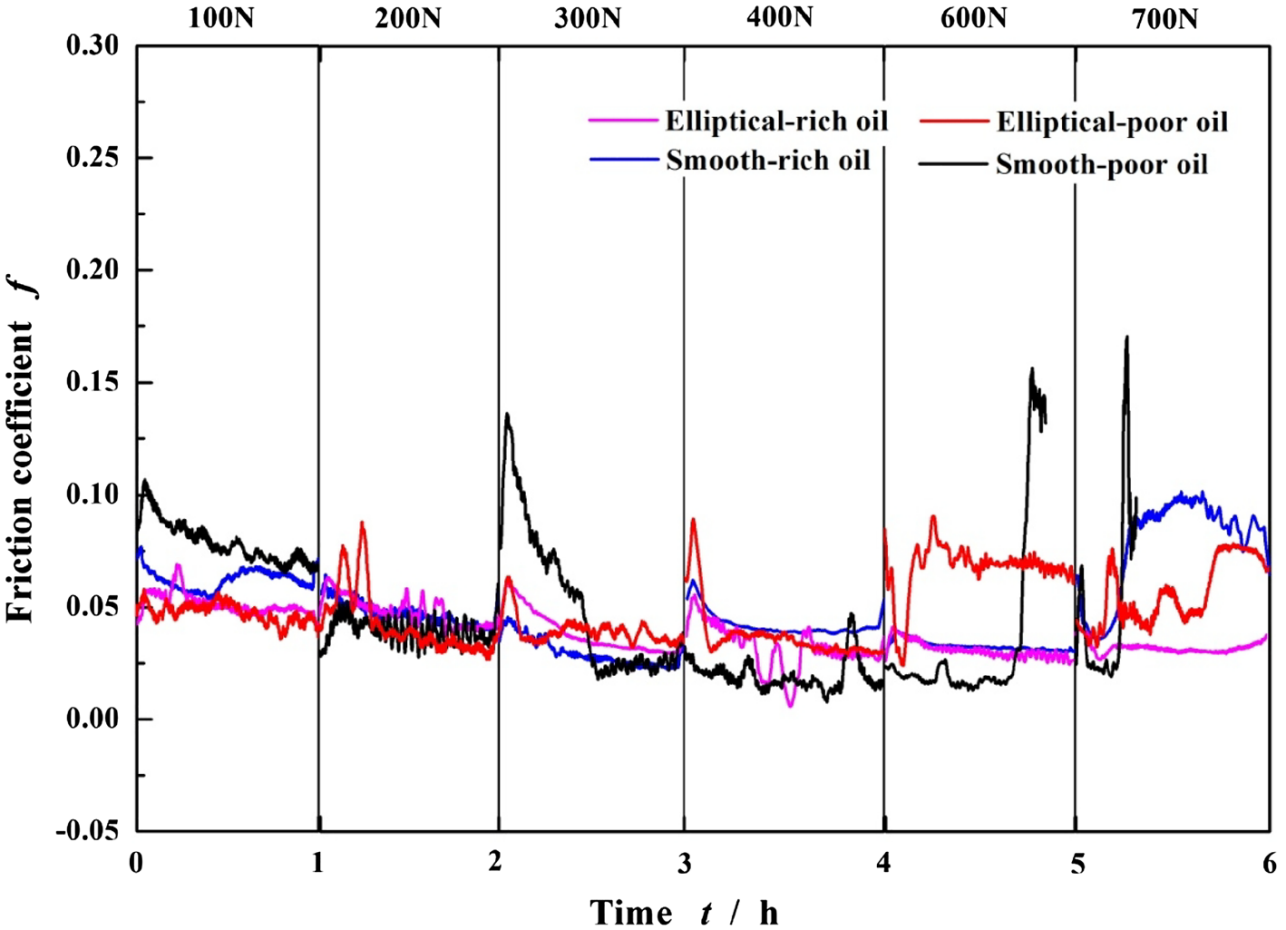
Friction coefficients of smooth surface and elliptical dimpled surface with the increase of time and loads under rich and poor lubrication conditions.

In the author’s last article^23^, the tribological behaviors under rich-oil condition have been analyzed. Figure 5 compares the friction coefficients of textured surface with the smooth one under rich-oil and poor-oil lubrication conditions with the increase of time and loads. It should be noted that each load condition represents a separate test, and then these obtained results are combined together into one figure. Each test with new specimens and lubricant is conducted from 0 to 60 min.

Generally speaking, to any case with the certain applied load, as the running time increases, the friction coefficient firstly increases rapidly during the start-up and acceleration phases from *ω* = 0 to 1500 r min^−1^, then decreases slowly while entering into the stable wear stage, finally maintains steady approximately. However, this has several special operating condition such as *F* = 700 N for the smooth surface under rich-oil lubrication and 600–700 N for the textured one under poor-oil lubrication. The friction coefficient* f* increases sharply before entering into the stable wear stage. Here, the friction interface occurs serious wear. Besides, to *F* = 600–700 N for the smooth surface under poor-oil lubrication, the *f* breaks the steady value and increases sharply along with significant vibration, indicating surface wear failure. As a result, the test is forced to end without running for 60 min.

With the load increasing from 100 to 700 N, to the textured surface with elliptical dimples, the friction coefficient presents a decreasing tendency under rich-oil lubrication. It is similar under poor-oil lubrication when *F* < 400 N, but the difference is that a minimum emerges at *F* = 400–500 N, then *f* increases with a larger amplitude. To the smooth surface, the lubrication conditions generate slight impact on the curve of *f*, just change the value instead of the tendency. As similar as the textured surface under poor-oil lubrication, their minimums are at about 300–400 N.

**Figure 6:**
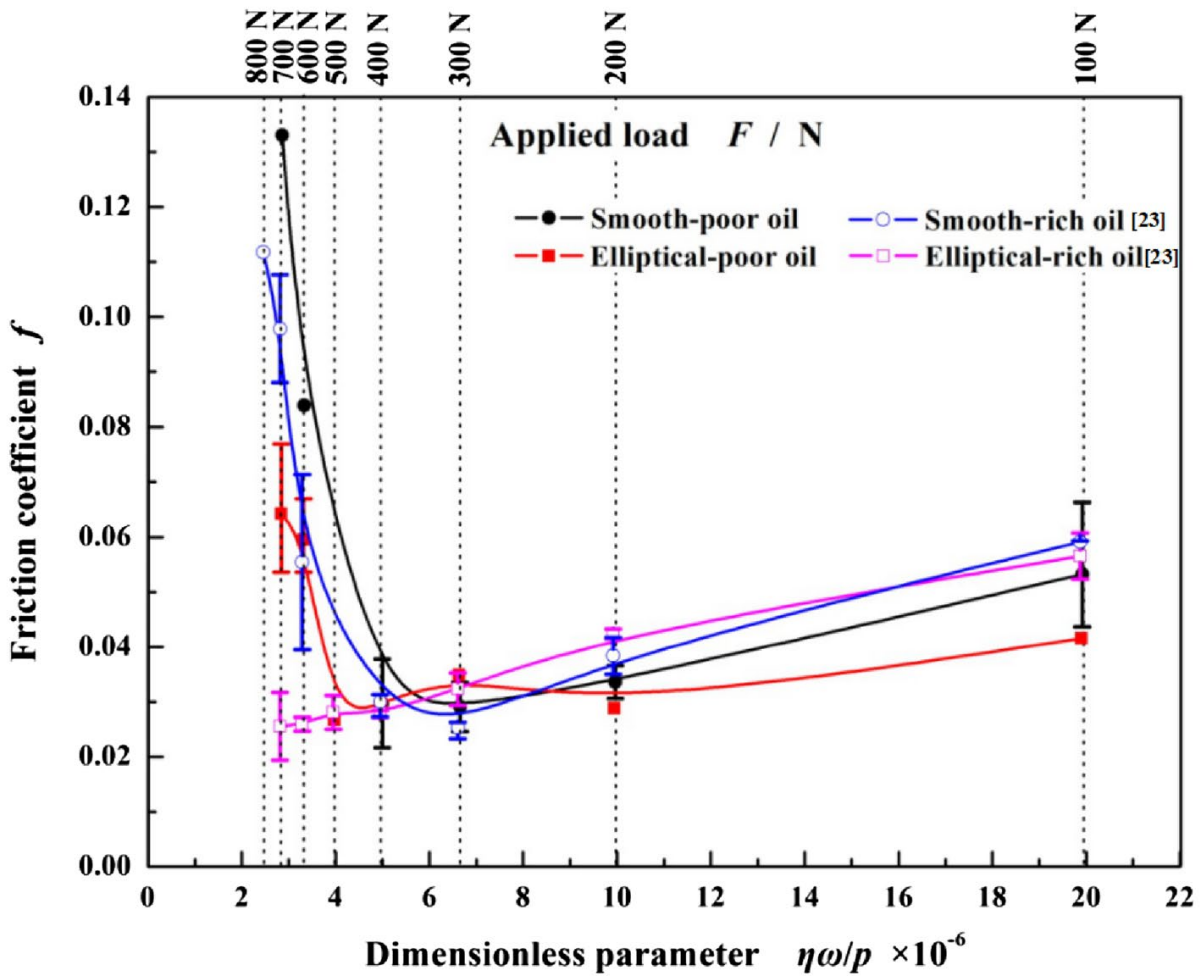
Friction coefficients of smooth and elliptical dimpled surfaces with the increase of dimensionless parameter under rich^23^ and poor lubrication conditions (*η* is the viscosity of the lubricant, *p*, the load per unit area and *ω*, the rotational speed).

The friction coefficients of steady phase (in Fig. 5) for the textured and smooth specimens under different applied loads and lubrication conditions are analyzed to form the Stribeck curves shown in Fig. 6. The dimensionless parameter *ηω*/*p* acts as the abscissa, the friction coefficient *f* acts as the ordinate. As the *ηω*/*p* decreases (right-to-left viewing), namely the applied load increases while *η* and *ω* keep constant, for the smooth surface under two lubrication conditions, the changes of *f* are pretty close. Results show that the *f* firstly decreases and reaches a minimum at *F* = 300 N, 0.025 for rich-oil lubrication and 0.029 for poor-oil lubrication respectively, then increases with a larger amplitude. This phenomenon illustrates the lubrication regime transforms form hydrodynamic into mixed lubrication near *F* = 300 N. When *F *< 300 N, the smooth surface is in hydrodynamic lubrication in which the poor-oil condition presents a smaller friction coefficient than the rich-oil one. Conversely, when *F *> 300 N under mixed regime, the rich-oil condition shows the better friction reduction advantages.

**Figure 7:**
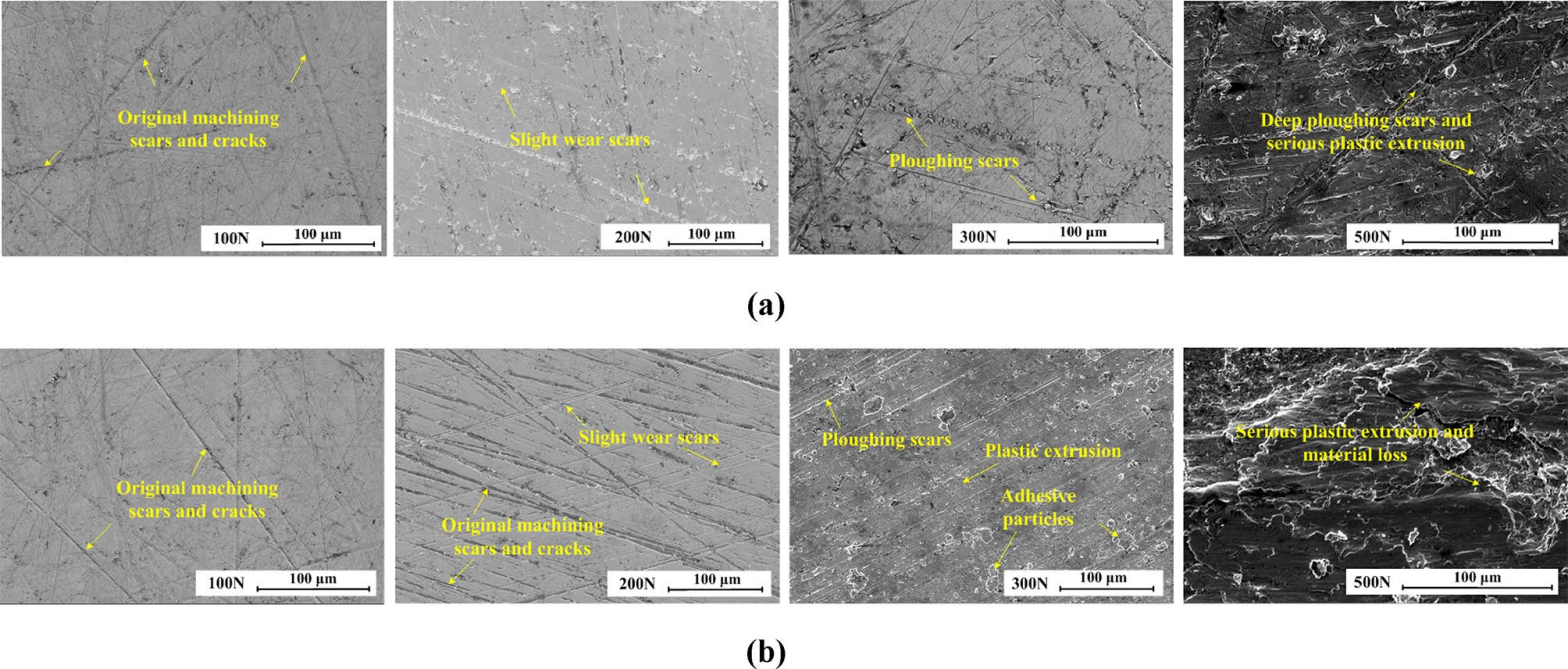
Wear topographies of bottom specimens versus the smooth upper specimens: (**a**) under rich-oil lubrication condition; (**b**) under poor-oil lubrication condition.

While the tests were completed, the wear topographies on frictional surfaces of bottom specimens were presented by SEM analysis under oil film lubrication conditions as shown in Fig. 7. To the surface versus the smooth upper specimen, a similar development trend of wear topographies can be found clearly. The surfaces distribute the widely original machining scars and cracks in *F* = 100 N, and the slight wear scars in *F* = 200 N. With the load increasing to 300 N, the ploughing scars induced by two-body abrasive wear and plastic extrusions induced by three-body abrasive wear are formed gradually. Besides, the partial adhesive particles and pits also can be existed under poor-oil condition. When the *F* reached beyond 500 N, the material loss owning to the deep ploughing scars and serious plastic extrusion becomes the main wear mode. Overall, When *F *< 300 N under hydrodynamic lubrication analyzed in Fig. 6, the poor-oil condition presents a similar wear resistance as the rich-oil one, and the abrasive wear is the dominant wear mechanism. When *F *> 300 N under mixed regime, the material loss induced by mechanical rubbing between the rotor and stator is the dominant wear mechanism, and the rich-oil condition presents a better wear resistance.

To summarize the smooth surface, when the friction interface is in hydrodynamic lubrication regime, it is not that the more lubricant, the better friction behavior. It just needs a certain amount of lubricant to ensure the continuous oil film. Here, this can obtain the lower friction power consumption, meanwhile maintain similar wear resistance as the rich-oil condition. But when in mixed lubrication regime, the more lubricant can guarantee the better friction reduce and wear resistance.

For the textured surface under poor-oil condition in Fig. 6, the *f* presents a similar tendency to that of smooth one. The dimples improve the critical load from 300 N for smooth surfaces to 400–500 N, thus extend the range of hydrodynamic regime, which contributes to enhance the tribological performance. And the minimum is about 0.030 closed to the smooth one. But for the rich-oil condition, the *f* decreases invariably with the increase of applied load and has no inflexion, which illustrates the textured surface always maintains hydrodynamic lubrication regime. To this, the reason may be attributed to that a significant hydrodynamic effect induced by elliptical dimples enhances the load carrying capacity of oil film to establish the steady hydrodynamic lubrication. Another is that the dimples can be served as a micro-reservoir to supply sustained lubrication under applied load^30–33^.

**Figure 8:**
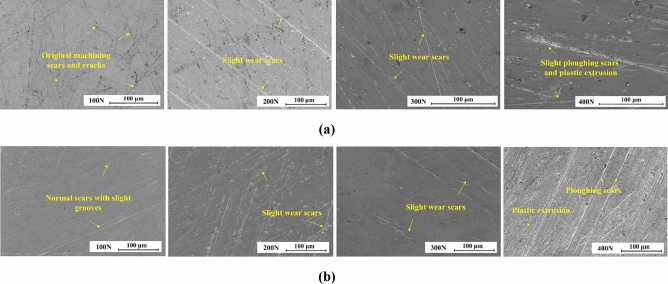
Wear topographies of bottom specimens versus the textured upper specimens: (**a**) under rich-oil lubrication condition; (**b**) under poor-oil lubrication condition.

Figure 8 shows the wear topographies of bottom specimens versus the textured upper specimens. Under rich-oil condition, the only slight wear scars and the original machining scars or cracks are presented. However, under poor-oil condition, the original machining features are disappeared after running the stable wear stage, as a result, the frictional interfaces become much smoother. When *F *> 400 N under mixed regime analyzed in Fig. 6, the slight ploughing scars and plastic extrusions are formed gradually. Compared with the smooth one, under the hydrodynamic regime, the laser surface texturing does not generate influences on wear resistance, but under the mixed regime, the wear resistance can be obtained significant improvement. The reason for this is that the dimples served as lubricant container to provide sufficient lubrication condition for the mating surfaces. While applying load, the micro dimples can maintain the stable oil film. The hydrodynamic effect enhances the load carrying capacity of oil film, thus makes the mating surfaces separate and keeps the non-contact running^34^.

In conclusion, compared with the smooth surface without textures under oil film lubrication, the textured surfaces with elliptical dimples can effectively improve the lubrication regime and enhance wear resistance, especially for rich-oil condition and high applied load because of the more significant hydrodynamic effect of elliptical dimples. In present case, the friction coefficient decreases significant when the applied load is greater than 500 N under rich-oil condition.

**Figure 9:**
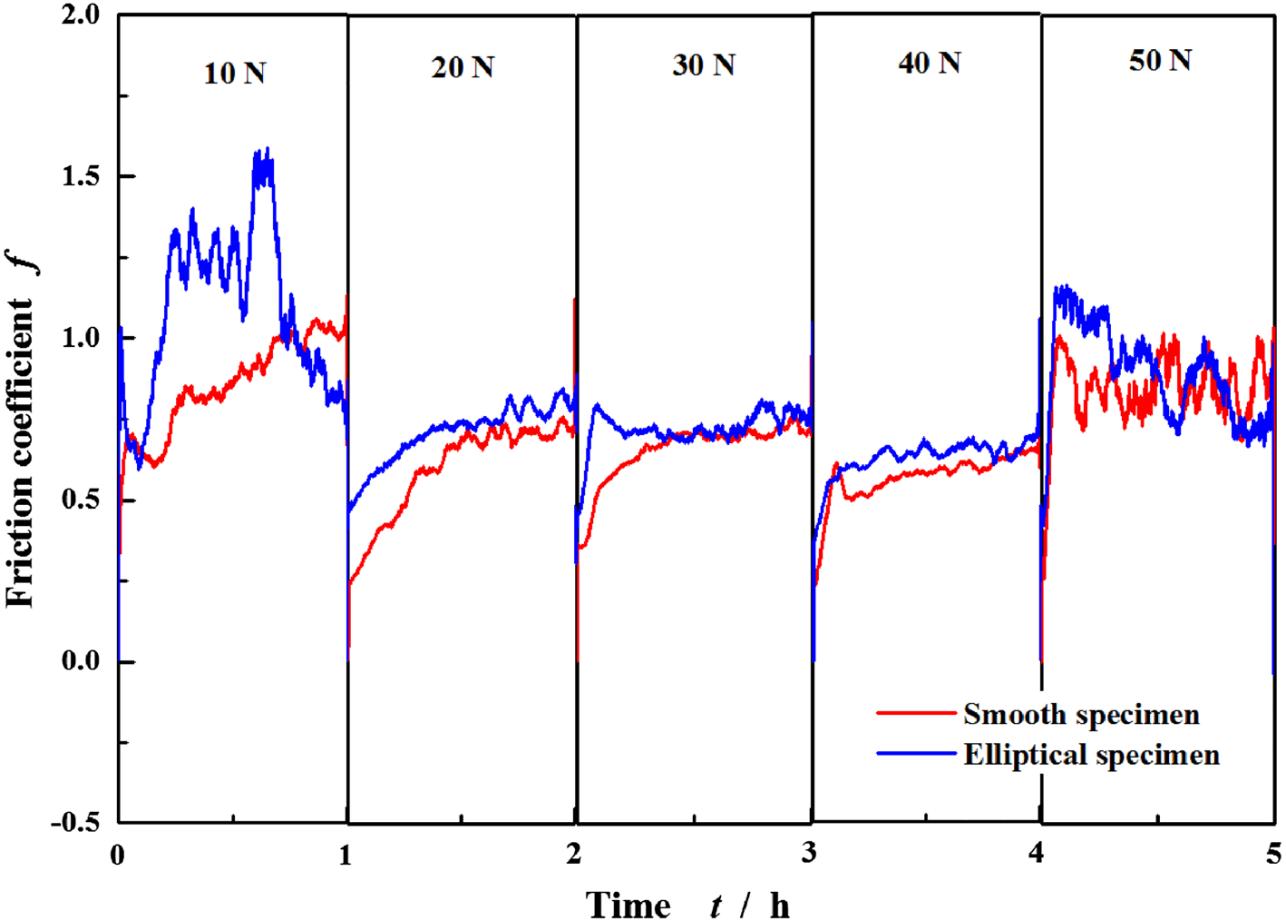
Friction coefficients of smooth surface and elliptical dimpled surface with the increase of time and loads under dry lubrication conditions.

Figure 9 shows the curves of friction coefficient of textured surface and smooth surface under dry lubrication conditions. When the applied load is controlled in 100–700 N, the friction interface fails sharply shortly with test start-up. So the load of 10–50 N is applied in present dry lubrication. It is found that the friction coefficient *f* significantly increases compared with the oil film lubrication, and the maximum can reach about 1.5. Besides, the *f* of textured surface is greater than that of the smooth one, which shows the dimpled textures have no the advantage of friction reduction and make the opposite effect. So the laser surface texturing is not recommended in dry lubrication condition if with the purpose of improving tribological behavior.

**Figure 10:**
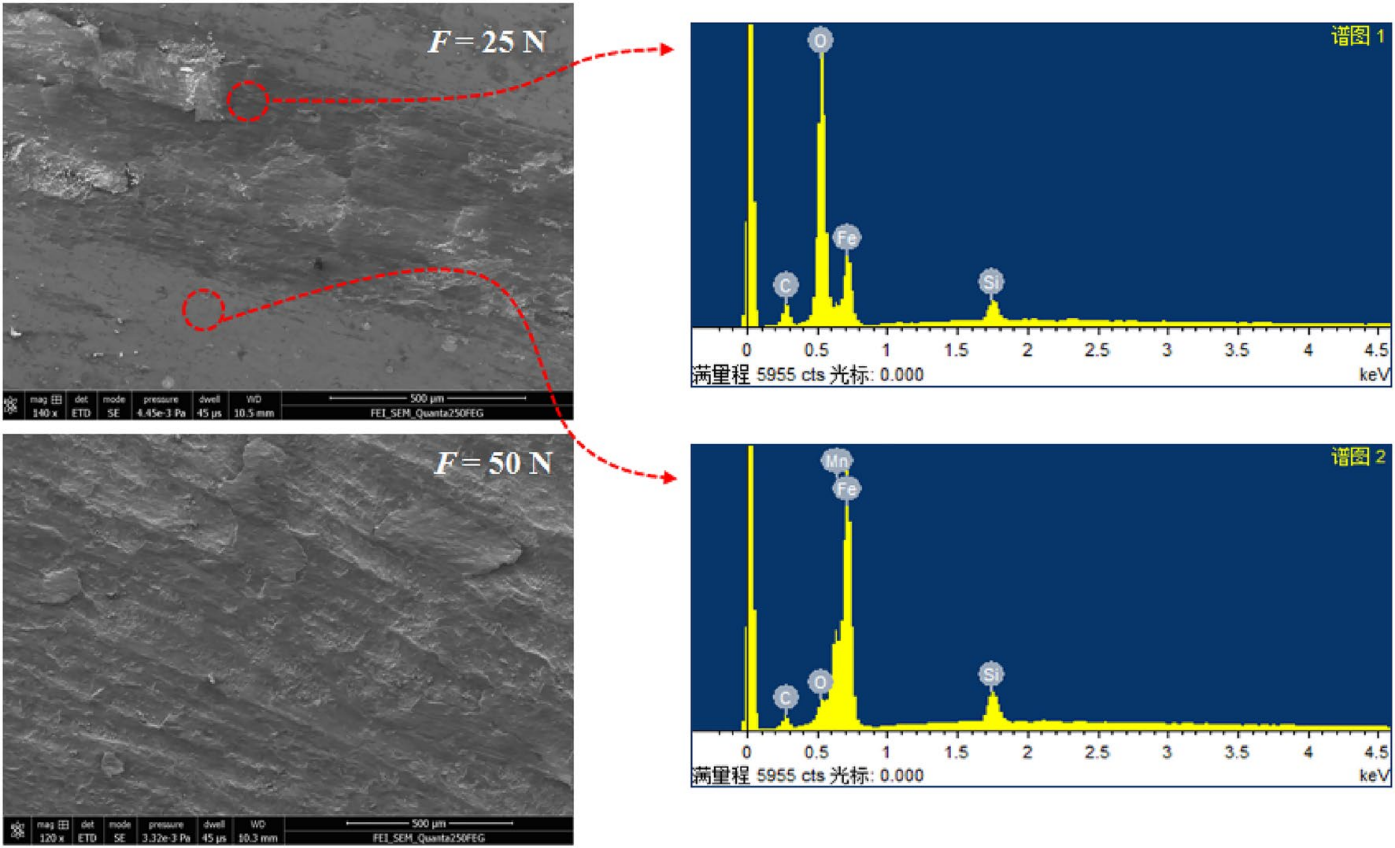
Wear topographies under dry lubrication condition.

Wear topographies under dry lubrication condition in Fig. 10 can be found that the frictional interface generates the serious oxidation wear by EDS analysis, and the wear severity is similar for the textured and smooth specimens. The oxides are easy to be peeled off under the large localized contact pressure due to the plastic extrusion by mechanical contact by SEM analysis.

## Compressor performance tests

**Figure 11:**
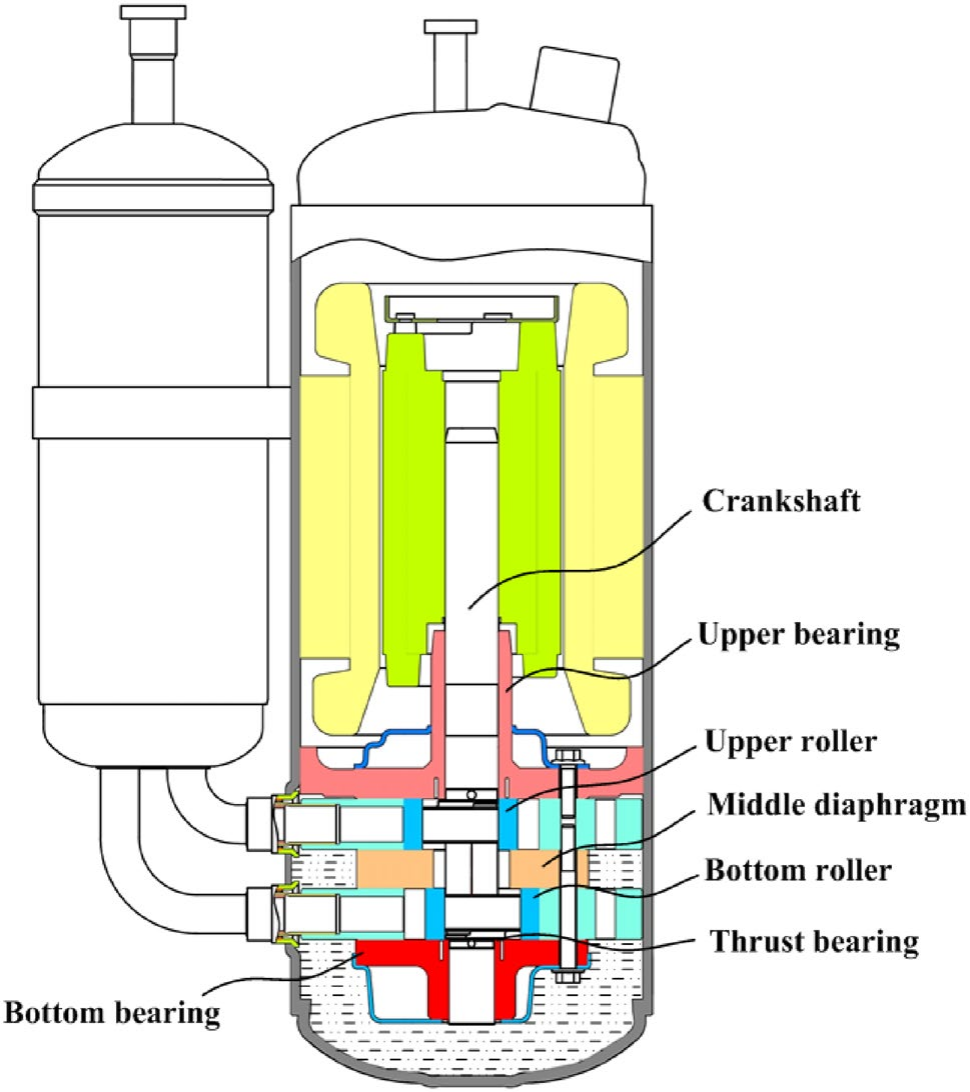
Schematic of test compressor.

By the above analysis, the surface textures can effectively decrease friction coefficient and enhance wear resistance especially for rich-oil condition and high applied load^26^. So the laser surface texturing is extremely suitable for the thrust bearing as shown in Fig. 11 to reduce the friction power consumption and improve the energy efficiency ratio.

**Figure 12:**
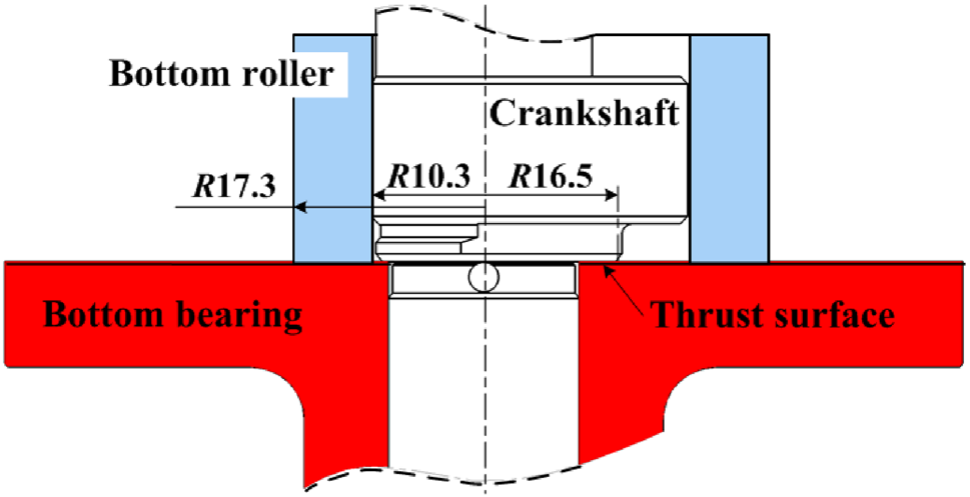
Trust surface.

**Figure 13:**
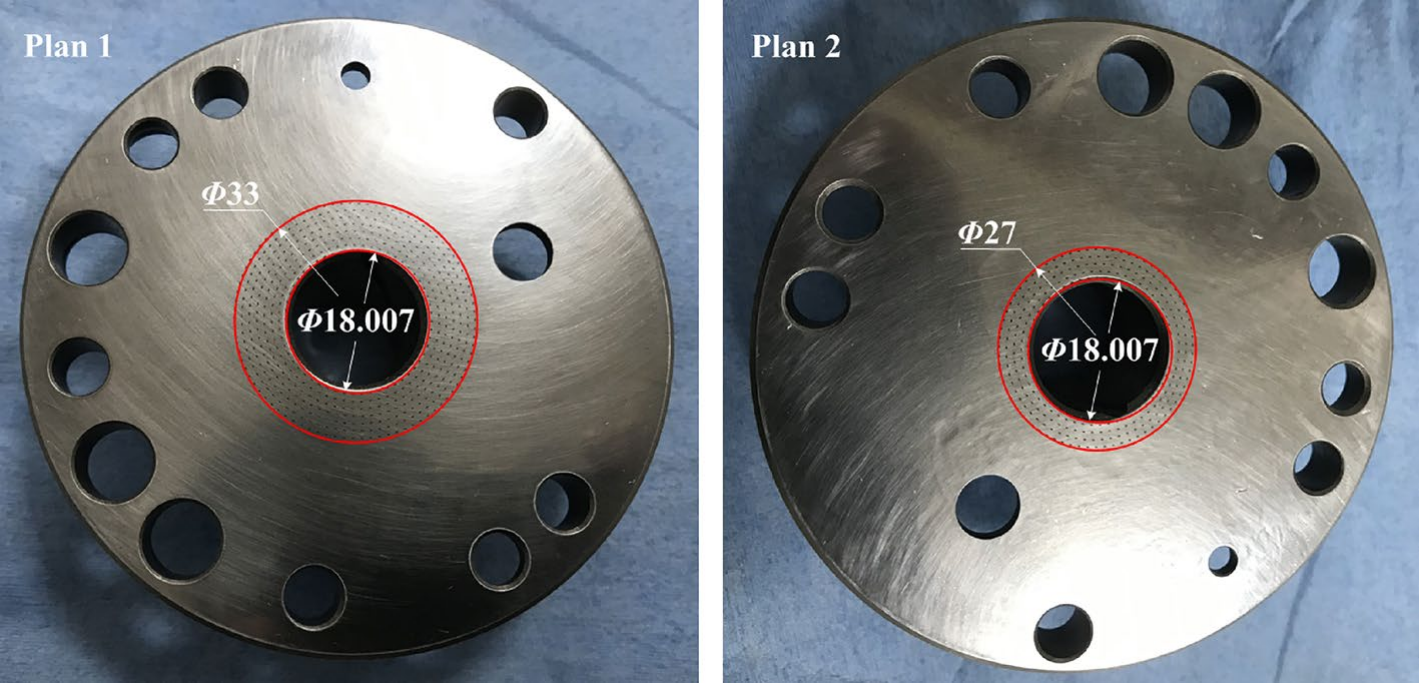
Photographs of bottom bearing with textures.

Figure 11 gives the schematic of rolling piston rotary compressor with two cylinders used to test. The contact interface between the bottom bearing and crankshaft is known as the thrust surface as shown in Fig. 12. On the surface of bottom bearing, the textures with ellipses are distributed with the same parameters as Table 1. The photographs of textured surfaces are shown in Fig. 13. Referring to two test plans, the distribution range of elliptical textures in ‘Plan 1’ is from *Φ*18.007 to *Φ*33 mm, and ‘Plan 2’, from*Φ*18.007 to *Φ*27 mm. Test conditions are listed on Table 3.

**Table 3:** Compressor operating conditions.

Item	Dimensions and data
Condensing temperature	65 °C
Evaporating temperature	13 °C
Subcooling degree	7 °C
Suction temperature	24 °C
Ambient temperature	35 °C
Frequency	60 Hz

The compressor test was conducted in Gree Electric Appliances, Inc. of Zhuhai (Guangdong Zhuhai, China) with the compressor performance test bench supported by Shanghai Tianhan Air-handling Equipment Co., Ltd. Performance parameters including the cooling capacity, input power, COP (= cool capacity/input power) and current for compressor with fixed frequency can be obtained.

**Figure 14:**
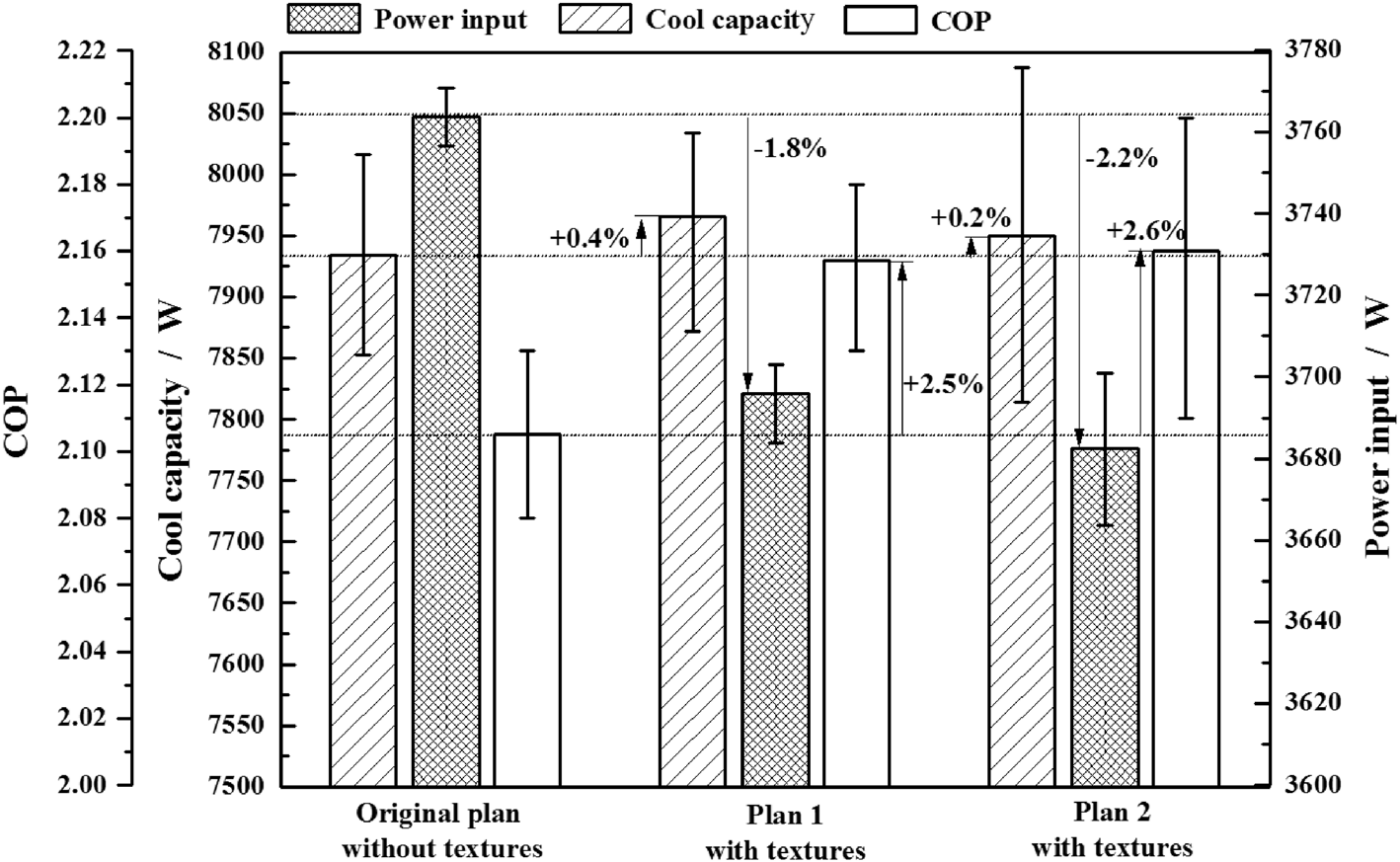
Comparisons of compressor performance for different plans.

A large number of compressor samples were tested to get this average performance value and weaken the influence of the measurement error. Three identical compressors for each plan with textures were tested with a comparison of original plan without textures. The test data was listed in Table 4. During testing process, the consistency of testing environment and the continuity of testing time.were ensured. Results in Fig. 14 show that textures fabricated on the thrust surfaces can significantly decrease the power input of compressor with the 1.8% reduction in Plan 1 and 2.2% in Plan 2. Meanwhile it has no influence on the cool capacity, which performs just 0.4% and 0.2% increase. As a result, the coefficient of performance (COP) can enhance 2.5% and 2.6% for Plan 1 and Plan 2 respectively. Besides, the two textured plans are effective to reduce the friction power consumption and improve the energy efficiency ratio, and have no obvious differences between them.

**Table 4:** Compressor performance test dada.

Test schemes	Compressor number	Cooling capacity/W	Input power/W	COP
Original plan without textures	NO. 01	7852.7	3770.9	2.08
	NO. 02	8016.3	3756.6	2.13
	NO. 03	7933.5	3768.8	2.11
	Average value	7934.2	3765.4	2.11
Plan 1# with textures	NO. 04	7871.8	3702.9	2.13
	NO. 05	8034.2	3683.8	2.18
	NO. 06	7990.5	3700.9	2.16
	Average value	7965.5	3695.9	2.16
Plan 2# with textures	NO. 07	8087.7	3682.7	2.20
	NO. 08	7946.6	3663.7	2.17
	NO. 09	7814.1	3700.9	2.11
	Average value	7949.5	3682.4	2.16

## Conclusions

The tribological benefits of laser textures were experimentally compared under different lubrication conditions by tribological tests, and verified by thrust surface for rolling piston rotary compressors. The lubrication regime and wear mechanism were discussed by friction coefficients and wear topographies. The following conclusions were drawn.

1. The tribological improvement by textured surfaces strongly depends on lubrication condition. With the increase of applied loads under rich-oil and poor-oil lubrications, the effect of micro dimple promotes the critical load transforming lubrication regime, and expands the range of hydrodynamic lubrication, meanwhile maintains a similar minimum of friction coefficient as the smooth surface but enhances wear resistance. However, it is reverse to increase the friction coefficient for the textured surfaces under dry lubrication.
2. The surface textures can effectively decrease friction coefficient and enhance wear resistance especially for rich-oil condition and high applied load because of the more significant hydrodynamic effect of elliptical dimples. But it is not recommended in dry lubrication condition if with the purpose of improving tribological behavior. The abrasive wear is the dominant wear mechanism under oil film lubrication, but oxidation wear under dry lubrication.
3. The compressor performance can be improved significantly by laser surface texturing, which is effective to reduce the friction power consumption and improve the energy efficiency ratio, and have no obvious influences on the cool capacity. In present cases, the power input of compressor can be decreased ~ 2%, and the coefficient of performance (COP) can enhance ~ 2.5%.

## Data Availability

All data generated or analyzed during this study are included in this published article.
